# Thermodynamic Study of the Sustainable Hydrometallurgical Treatment of Copper Converter Flue Dust Based on Pb, Zn, and Sn Oxides

**DOI:** 10.3390/ma17235690

**Published:** 2024-11-21

**Authors:** Alexandra Kollová, Martina Laubertová, Jarmila Trpčevská, Martin Sisol

**Affiliations:** 1 Institute of Recycling Technologies, Faculty of Materials, Metallurgy and Recycling, Technical University of Košice, Letná 1/9, 042 00 Košice, Slovakia; alexandra.kollova@tuke.sk (A.K.); jarmila.trpcevska@tuke.sk (J.T.); 2 Institute of Earth Resources, Faculty of Mining, Ecology, Process Control and Geotechnologies, Technical University of Košice, Letná 1/9, 042 00 Košice, Slovakia; martin.sisol@tuke.sk

**Keywords:** flue dust, thermodynamic study, leaching, hydrometallurgy, recycled material

## Abstract

The presented article deals with the thermodynamic study of copper converter flue dust (CCFD) treatment by hydrometallurgical process. The investigated sample of CCFD contains 38.31 wt.% Zn, 11.35 wt.% Pb, and 2.92 wt.% Sn in the form of oxides (PbO, ZnO, and SnO_2_). The leaching of CCFD in sulphuric acid, acetic acid, nitric acid, and sodium hydroxide was thermodynamically studied. Using Pourbaix diagrams for individual metal–S/C/N/Na/–H_2_O systems, the possibility of leaching oxides in selected leaching agents was confirmed. A sustainable and environmentally friendly method of processing the sample of CCFD using a hydrometallurgical method is proposed. A suitable selective leaching agent is the acetic acid solution. When leaching in an acetic acid solution, zinc and lead are chemically dissolved to form acetates Zn(CH_3_COO)_2(aq)_ in the form of Zn^2+^_(aq)_ at a temperature of 20 °C with a pH range of 0–3.5 and at a temperature of 80 °C with a pH range of 0–2.95, as well as Pb(CH_3_COO)_2(aq)_ as Pb^2+^_(aq)_ at a temperature of 20 °C with a pH range of 0–1.95 and at a temperature of 80 °C with a pH range of 0–2, respectively, while tin remains as a solid residue in the form of SnO_2(s)_ at the temperatures of 20 °C and 80 °C throughout the whole acidic pH range. Various analytical techniques, such as SEM, EDX, XRD, and AAS, were used to analyse samples before a thermodynamic study of the leaching of CCFD was conducted by applying Pourbaix Eh–pH diagrams.

## 1. Introduction

With a rapid increase in the demand for copper, mainly for information technology and electromobility, and with an increase in the copper production capacity, there has been an increase in the production of this metal. Currently, copper is considered the “new gold”. Copper is currently produced primarily by pyrometallurgical processes (about 84%), with 67% of refined copper coming from pyrometallurgical production from primary raw materials, which are sulphide ores. About 16% of copper is produced hydrometallurgically, mainly from lower-quality oxidic ores and some sulphide ores. In 2023, 26.5 million tonnes of refined copper were produced in the world [1,2]. In the pyrometallurgical production of copper, depending on the operating conditions, two basic methods are used: flash smelting and bath smelting. About half of the total amount of Cu matte is produced by flash smelting. The products produced by this process are matte, slag, and off-gases. Two types of flash-smelting processes are used: the Metso Outotec (formerly Outokumpu) process (44 furnaces in operation) and the Inco process (two furnaces in operation) [3,4,5]. The largest companies producing refined copper cathodes are Boliden AB, Rönnskär, Sweden; Aurubis AG, Lünen, Germany; Umicore Precious Metals Refining NV/SA, Hoboken, Belgium; Glencore PLC, Baar, Switzerland; and Montanwerke Brixlegg AG, Brixlegg, Austria, which owns Kovohuty, a. s., Krompachy, Slovakia [6].

**Figure 1:**
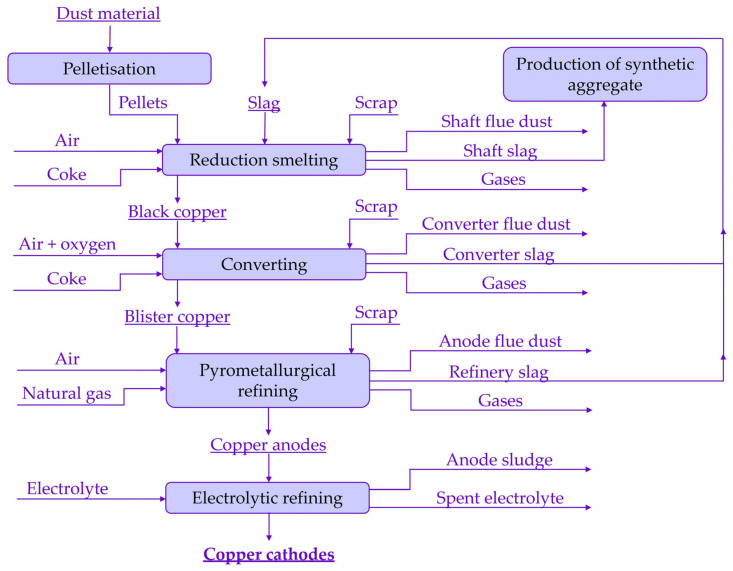
Technological diagram of copper production in Kovohuty, a. s., Krompachy, Slovakia.

Kovohuty, a. s., Krompachy, Slovakia, is the only metallurgical company based in Slovakia, where copper production has a long history and tradition, namely the recycling of waste with different copper contents. The main product is copper anodes (containing 99% Cu), which are processed at the parent company Montanwerke Brixlegg AG, Brixlegg, Austria. The technological scheme of copper production in Kovohuty, a. s., Krompachy, Slovakia, is shown in Figure 1 [7,8].

When processing secondary raw materials containing copper, hydrometallurgical processes and bath smelting are used. The method of processing raw materials depends on the Cu content, the nature of the material, and the amount of material [9]. In recent years, due to the depletion of primary raw materials, copper ores have been degrading, which leads to an increased generation of waste from individual processes. The main solid wastes arising from pyrometallurgical copper production are slags and flue dusts. Flue dust from copper production can be defined as a fine-grained product obtained from the melt in the form of dust, which is sucked by fans through filter stations and subsequently captured in electrostatic precipitators or fabric filters. The direct use of these dusts to produce copper is not convenient due to the high content of metals such as As, Pb, Cd, and Bi. These metals cause significant technological problems in the direct pyrometallurgical processing of flue dusts. These metals usually enter the production cycle of copper through the processing of copper scrap materials, such as brass, bronze, WEEE (waste from electrical and electronic equipment), etc. Moreover, copper flue dusts are considered hazardous waste according to the European Waste Catalogue (codes 10 06 03 flue gas dust and 10 06 06 solid wastes from gas treatment) because of the high content of toxic heavy metals and the fine-grained character of flue dusts [10,11,12].

These dusts are investigated in laboratory conditions using mainly hydrometallurgical procedures. The article aims to provide a literary overview of the processing of flue dusts from copper production and to study the thermodynamics of hydrometallurgical processing of the converter flue dust generated during the secondary production of copper in a Slovak company.

Copper production flue dust is defined as the product obtained from off-gases found in furnaces, pipelines, and settling chambers due to roasting, smelting, converting, and pyrometallurgical refining operations. The amount of this dust can represent 2–15 wt.% of the incoming concentration. During different smelting and converting processes, different amounts of flue dust are produced. Copper flue dust (CFD) is captured in electrostatic separators or fabric filters. The formation of flue dust from copper production is due to mechanical and physical-chemical reasons. By blowing the compressed air supplied by the blowers, small particles of the charge, slag, and metallic copper are removed from the converter. At the same time, vapours of volatile oxides of Pb, Zn, and Sn are entrained by the gases, which condense into a solid state upon cooling and are captured in the filter device [13,14]. Among the elements found in copper flue dusts are, for example, Cu, Zn, Fe, Pb, Ni, and As. CaO, MgO, Al_2_O_3_, and SiO_2_ come from slag-forming additives and furnace lining. Flue dust from secondary copper production may also contain Cl and Br, which originate from organic residues in the charge materials of secondary copper smelters, such as plastics from e-waste, cables, and polyvinyl chloride (PVC) [15,16]. The composition of the dust from copper production is influenced by the composition of the charge (mineralogy of concentrates, slag-forming additives, returnable material, and the weight ratio of the charge components), process conditions (temperature, oxidation-reduction conditions, and duration), and the consistency of metal distillation to the gas phase or its intensity [17,18,19]. It is a frequent practice to reversibly recycle flue dusts in smelters to obtain copper. However, by recycling flue dusts in this way, the capacity of the furnace is reduced, and thus, the productivity. The energy consumption of smelting processes is increasing. Another problem is represented by impurities such as As, Sb, Bi, and Pb, which accumulate in the matte, in the converter copper, and in the anode copper by recycling the flue dust in the aforementioned way. This makes electrolytic refining difficult. Many hydrometallurgical and pyrometallurgical processes have been proposed for copper flue dusts treatment [4,13]. Companies processing flue dusts from copper production include, e.g., Jean Goldschmidt International S.A., Brussels, Belgium; Metaal Magnus International B.V., Amstelveen, Netherlands; and Eco-Zinder S.p.A., Trezzo sull’Adda, Italy [20,21,22]. The activities of Jean Goldschmidt International S.A., Brussels, Belgium, are focused on the processing of a wide range of complex residues, by-products, and raw materials containing non-ferrous metals. The hydrometal plant was founded in 2021. Its activity is focused on the processing of complex Zn-containing materials to produce zinc salts by hydrometallurgical processes [20]. In 2020, Jean Goldschmidt International S.A., Brussels, Belgium, and Zinc Nacional S.A., San Nicolás de los Garza, Mexico, Nuevo León, created Harz Oxid GmbH, Goslar, Germany, a joint venture in which both companies operate the zinc recycling business of Harz-Metall GmbH, Goslar, Germany. Charged materials containing zinc are processed by the Waelz kiln process to produce Waelz oxides with a zinc content of at least 56%. The Waelz kiln process takes place in a rotary kiln with a length of 40 m at a temperature of approximately 1200 °C in the presence of additives. Waelz oxides are then distributed to major zinc refineries in Europe [20,23]. Metal Magnus International B.V., Amstelveen, The Netherlands, provides recycling of various non-ferrous and precious metal by-products. These materials include catalysts, ashes, concentrates, slags, flue dusts, filter cakes, high-grade alloy scrap, sludges, and other residues. MMI annually purchases thousands of tons of materials produced in petrochemical and manufacturing plants around the world. MMI works directly with smelters, refineries, and other companies focused on the reuse or processing of valuable commodities [21]. Eco-Zinder S.p.A., Trezzo sull’Adda, Italy, was founded in 1971. Since 1987, Eco-Zinder S.p.A., Trezzo sull’Adda, Italy, has been one of the main Italian companies involved in the production of raw materials from industrial waste. Eco-Zinder S.p.A., Trezzo sull’Adda, Italy, is part of the ITALBIOTEC consortium and the Lombardy Green Chemistry Association. The main products of Eco-Zinder S.p.A., Trezzo sull’Adda, Italy, are zinc salts (zinc carbonates) and concentrates derived from them, which are intended mainly for metallurgical plants. The zinc salts and concentrates are raw materials containing zinc (40%) and copper (20%) in the form of sulphates [24,25].

Before designing a process of a converter dust sample treatment, it is important to know the methods of processing copper flue dusts on an industrial scale and to devote laboratory research to the processing of copper flue dust, especially in the field of hydrometallurgy.

### 1.1. Laboratory Research on the Processing of Flue Dusts from Copper Production

The acidic leaching of CFD in H_2_SO_4_ solution at a constant pH was studied (Equation (1)) [19,26,27]. After the precipitation of tin and iron, copper was obtained by zinc cementation. By adding Na_2_CO_3_, zinc was precipitated from the solution (Equation (2)) and the precipitate was calcined to ZnO (96.16% Zn) (Equation (3)) at a temperature of 900 °C [26].

(1) $${ZnO}_{\left(s\right)}+{H_{2}SO_{4}}_{\left(aq\right)}={ZnSO_{4}}_{\left(aq\right)}+{H_{2}O}_{\left(l\right)}$$

(2) $${ZnSO_{4}}_{\left(aq\right)}+{{Na}_{2}CO_{3}}_{\left(s\right)}={ZnCO_{3}}_{\left(s\right)}+{{Na}_{2}SO_{4}}_{\left(aq\right)}$$

(3) $${ZnCO_{3}}_{\left(s\right)}={ZnO}_{\left(s\right)}+{CO_{2}}_{\left(g\right)}$$

In the work, authors [28] dealt with the pressure leaching of copper smelting dust in a H_2_SO_4_ solution at a temperature of 453 K (179.85 °C). Copper, zinc, iron (Equation (4)), and arsenic (Equation (5)) were leached from the flue dust. The addition of Fe^2+^ ions did not change the leaching of zinc, and the leaching of copper was affected only slightly, while the leaching of arsenic was completely inhibited. Coprecipitation of arsenic with iron occurred. Other metals, such as bismuth, lead, tin, and antimony, remained in the solid residue.

(4) $$MeS+H_{2}SO_{4}+1/2O_{2}=MeSO_{4}+S^{0}+H_{2}O(Me:Cu,Zn,Pb)$$

(5) $${As}_{2}S_{3}+3H_{2}O+5/2O_{2}=2H_{3}AsO_{4}+3S^{0}$$

The authors [27] used liquid extraction of Cu from an acidic solution using LIX 860 or MOC-55TD oximes (Equation (6)) to treat CCFD. Electrolyte from copper electrolysis was used for copper stripping. By using LIX 860, a solution richer in copper was obtained, and stripping from MOC-55TD was easier.

(6) $${{Cu}^{2+}}_{\left(aq\right)}+2{HR}_{\left(org\right)}↔{CuR_{2}}_{\left(org\right)}+2{H^{+}}_{\left(aq\right)}$$

Converter flue dust by leaching in a NaOH solution at ambient temperature (Equation (7)) and reductive smelting of the solid residue with soda were studied. As the temperature, the amount of reducing agent, and soda increased, the yield of metals increased, but there was also an increase in the concentration of Cu and Ag in the metal phase [29].

(7) $$MeSO_{4}+2NaOH\to Me{\left(OH\right)}_{2}+{Na}_{2}SO_{4}$$

The authors of [30,31] leached secondary CCFD in (NH_4_)_2_CO_3_–NH_4_OH, while ZnO, Zn, and partially Cu, Pb, and Sn passed into the solution. After that, a cementation precipitate copper was obtained by zinc cementation. Precipitation with CO_2_ and roasting Zn(NH_3_)_2_CO_3_ · ZnO at 400 °C resulted in 99% ZnO. The authors [13] proposed two ways of processing three types of copper flue dusts as follows: from the flash furnace, from the rotary furnace, and from the converter. The first procedure involved leaching flue dusts in ammonia-based leaching agents (Equation (8)), liquid extraction of LIX 860, LIX 84, or LIX 54 (Equation (9)), and copper electrolysis. The second procedure consisted of leaching in ammonia-based leachers and copper cementation with zinc (Equation (10)). A copper recovery of over 98% was achieved.

(8) $$CuO+4NH_{3}+H_{2}O\to {Cu{\left(NH_{3}\right)}_{4}}^{2+}+2{OH}^{-}$$

(9) $${{Cu{\left(NH_{3}\right)}_{4}}^{2+}}_{\left(aq\right)}+2{HR}_{org}↔{CuR_{2}}_{\left(org\right)}+2{{NH_{4}}^{+}}_{\left(aq\right)}+2{NH_{3}}_{\left(aq\right)}$$

(10) $${Cu}^{2+}+{Zn}^{0}\to {Cu}^{0}+{Zn}^{2+}$$

Other authors [32] leached CCFD in H_2_SO_4_ with the addition of MnO_2_ concentrate, which acted as an oxidising agent (Equation (11)). The solid residue was then leached in NaCl solution and the solid residue from salt leaching proceeded to the second stage of salt leaching. The solutions from the first and second salt leachings (containing PbCl_2_) were mixed and calcined with the addition of soda ash at 550–570 °C to obtain PbO. PbO was subjected to reduction smelting with fluxes and graphite to obtain lead metal (99.87% Pb).

(11) $$MeO+H_{2}SO_{4}=MeSO_{4}+H_{2}O(Me:Cu,Zn,Pb)$$

Copper smelting flue dust was leached in a NaOH solution, and then the sulphides with Na_2_S were precipitated (Equations (12)–(14)). The leaching rates were 92.4% As, 36.9% Pb, and 13.4% Zn. More than 99% of lead and zinc precipitated from the leachate [33].

(12) $${As}_{2}O_{3}+2NaOH=2NaAsO_{2}+H_{2}O$$

(13) $$MeO+2NaOH+H_{2}O={Na}_{2}MeO_{2}+2H_{2}O(Me:Zn,Pb)$$

(14) $${Na}_{2}MeO_{2}+{Na}_{2}S+2H_{2}O=MeS↓+4NaOH(Me:Zn,Pb)$$

Converter flue dust from secondary copper production in acetic acid solution (Equation (15)) at an ambient temperature was leached, and the lead by zinc from the leachate was cemented (Equation (16)). Almost a 90% lead removal efficiency was achieved. The cementation precipitate contained lead in the form of PbO, Pb_5_O_8_, and Pb(Cu_2_O_2_), and copper.

(15) $${2CH_{3}COOH}_{\left(aq\right)}+{MeO}_{\left(s\right)}={Me{\left(CH_{3}COO\right)}_{2}}_{\left(aq\right)}+{H_{2}O}_{\left(l\right)}(Me:Pb,Zn)$$

(16) $${Zn}_{\left(s\right)}+{Me{\left(CH_{3}CO_{2}\right)}_{2}}_{\left(aq\right)}={Zn{\left(CH_{3}COO\right)}_{2}}_{\left(aq\right)}+{Me}_{\left(s\right)}(Me:Pb,Cu)$$

Liu et al. [34] mechanically pretreated the copper smelting flue dust and leached it in various leaching agents. The best results were obtained using a mixed acid solution (H_2_SO_4_ + HCl) and H_2_O_2_. The main leaching reactions, where H_2_O_2_ acts as an oxidising agent, are represented by Equations (17)–(21). Under optimal conditions, copper, arsenic, and iron leaching efficiencies of 95.27%, 96.82%, and 46.65% were achieved.

(17) $$CuO+2H^{+}\to {Cu}^{2+}+H_{2}O$$

(18) $${Fe}_{2}O_{3}+6H^{+}\to 2{Fe}^{3+}+3H_{2}O$$

(19) $$H_{2}O_{2}+2H^{+}+2e^{-}\to 2H_{2}O$$

(20) $$CuS+H_{2}O_{2}+2H^{+}\to {Cu}^{2+}+S+2H_{2}O$$

(21) $${As}_{2}O_{3}+H_{2}O+2H_{2}O_{2}\to 2H_{3}AsO_{4}$$

Authors [35] had leached shaft furnace dust in NaOH solutions to lead recovery. They discovered that a 2 M NaOH solution is optimal for obtaining 60% of lead (Equations (22) and (23)), and a 4 M NaOH solution is most suitable for obtaining 58% of zinc (Equation (24)).

(22) $$PbO+NaOH\to {Na}^{+}+{HPbO_{2}}^{-}$$

(23) $$PbSO_{4}+2{Na}^{+}+3{OH}^{-}\to {HPbO_{2}}^{-}+{Na}_{2}SO_{4}+H_{2}O$$

(24) $$ZnO+2NaOH\to {ZnO_{2}}^{2-}+2{Na}^{+}+H_{2}O$$

Ebrahimpour et al. [36] subjected copper smelting flue dust to acid leaching in the presence of three microbial cultures (mesophiles, moderate thermophiles, and extreme thermophiles). Up to 83% of copper from the flue dust was leached by a combination of three-phase bioleaching and chemical leaching in 6 days. The authors of the study [37] recycled flash furnace flue dust from primary copper smelting. The authors proposed to process the dust first by leaching in a NaOH solution, which would result in arsenic passing into the leachate, and then leaching into a H_2_SO_3_ solution, in which copper would pass into the leachate and bismuth would remain in the solid residue. Hydrometallurgically recovering bismuth from CCFD was investigated [38]. By leaching the flue dust in H_2_SO_4_ with the addition of NaCl (Equations (25) and (26)) it achieved 92% efficiency under optimal conditions. A conversion of BiCl_3_ to BiOCl by hydrolysis is described by Equations (27)–(29). Bi_2_O_3_ was obtained by mixing crude BiOCl cake with NaOH (Equation (30)) (100 g/L). The purity of the Bi_2_O_3_ product was approximately 97.8%, with arsenic, lead, and antimony being the main impurities.

(25) $${{Bi}_{2}O_{3}}_{\left(s\right)}+3H_{2}SO_{4}\to {{Bi}_{2}{\left(SO_{4}\right)}_{3}}_{\left(s\right)}+3H_{2}O$$

(26) $${{Bi}_{2}{\left(SO_{4}\right)}_{3}}_{\left(s\right)}+6NaCl\to {2Bi{Cl}_{3}}_{\left(aq\right)}+3{Na}_{2}SO_{4}$$

(27) $$Bi{Cl}_{3}+H_{2}O=Bi\left(OH\right){Cl}_{2}+HCl$$

(28) $$Bi{Cl}_{3}+2H_{2}O=Bi{\left(OH\right)}_{2}Cl+2HCl$$

(29) $$Bi{\left(OH\right)}_{2}Cl=BiOCl+H_{2}O$$

(30) $$2BiOCl+2NaOH+Heat\to {Bi}_{2}O_{3}+2NaCl+H_{2}O$$

By applying microwave and conventional leaching to flue dust from copper smelting, the authors [39] obtained copper and zinc. For leaching, they used a H_2_SO_4_ solution with/without the addition of HNO_3_. HNO_3_ acted as a copper oxidising agent and ZnO leaching agent. The total yield of copper was 69.83% in conventional leaching and 80.88% in microwave leaching.

The authors of [16] processed flue dust from secondary copper smelting. By roasting the flue dust, more than 99% of chlorine and bromine were removed (Equations (31)–(35)). Then, copper, zinc, and cadmium were almost completely dissolved in water. Lead was concentrated in the solid residue. In the last step, 96.7% of PbSO_4_ was reduced to elemental lead by mechanochemical reduction with iron powder at an ambient temperature.

(31) $$Pb{Cl}_{2}+H_{2}SO_{4}=PbSO_{4}+2HCl$$

(32) $$Pb{Br}_{2}+H_{2}SO_{4}=PbSO_{4}+2HBr$$

(33) $$Zn{Cl}_{2}+H_{2}SO_{4}=ZnSO_{4}+2HCl$$

(34) $$Zn{Br}_{2}+H_{2}SO_{4}=ZnSO_{4}+2HBr$$

(35) $$2CuBr+3H_{2}SO_{4}=2CuSO_{4}+SO_{2}+2HBr+2H_{2}O$$

### 1.2. Conclusion of Laboratory Research on the Processing of Flue Dusts from Copper Production

Copper flue dusts are primarily treated by hydrometallurgical methods. Hydrometallurgical processing of flue dust consists of multi-stage leaching (acidic or alkaline), refining of leachates, and obtaining saleable products from leachates by extraction methods. In pyrometallurgical treatment, reduction with subsequent evaporation of zinc and lead could be used. Since it is a fine-grained material, flue dust must be transported in closed containers or pretreated by palletisation. A small grain size can be advantageous in hydrometallurgical processing since increasing the reaction surface increases the rate of leaching. Also, from an environmental point of view, hydrometallurgy has an advantage, namely that there are no problems with off-gases, dust, and noise. Pyrometallurgical methods are more suitable for larger grains and more homogeneous materials. They are also less selective compared to hydrometallurgical methods. In pyrometallurgical processes, material could be lost during charging and during the process itself.

For copper flue dust leaching, most often, the H_2_SO_4_ solution, sometimes with the addition of NaCl or HNO_3_, is used. The NaOH solution, H_2_SO_3_ solution, solutions of leaching agents based on NH_3_, as well as mixed acidic and basic solutions, are also used for leaching. The literature review shows that the efficiency and mechanism of leaching depend on the chemical and phase compositions of the dust, the ratio of the solid and liquid phase, the temperature, the leaching time, the velocity of stirring, the type of leaching agent, and on the oxidation-reduction conditions. Some authors used mechanical and/or chemical treatment of the dust before leaching. The chemical composition of CFD depends on the composition of the charge, operating conditions, and the degree of metal distillation to the gas phase. Flue dust generated in different companies, therefore, has different compositions. A suitable processing method should be designed for each flue dust separately.

The goal was to study the thermodynamic conditions of leaching CCFD to find a selective leaching medium for the extraction of Pb, Zn, and Sn into the solution (leachate). Pourbaix Eh–pH diagrams were applied to predict whether a given chemical heterogeneous reaction will take place spontaneously in the direction of the formation of products after leaching.

## 2. Materials and Methods

In the first phase of the experimental part, the input sample, which comes from a Slovak company (Krompachy, Slovakia), was characterised. From a given batch of copper production, flue dust was provided, from which a representative sample was obtained by multiple quartering and homogenisation. The weight loss by drying was determined by drying a 10 g sample of the flue dust at a temperature of 110 °C for 2.5 h. The weight loss by annealing was determined by annealing 1 g of the sample at a temperature of 1000 °C for 1.0 h. The initial particle size of the flue dust sample was performed by laser diffraction using a Malvern Master-sizer 2000E (Malvern Instruments, Great Malvern, UK), accuracy: ±1%) with a Scirocco2000M dry sample dispenser (Malvern Instruments, Great Malvern, UK). The density of the sample was determined with a Micromeritics AccuPyc II 1340 gas pycnometer (Micromeritics Instrument Corporation, Norcross, GA, USA, accuracy: ±0.02% of the nominal cell volume). Chemical analysis of the sample was determined by the atomic absorption spectrometry (AAS) method for the elements copper, nickel, zinc, lead, iron, cadmium, and bismuth on a Varian Spectrophotometer AA20+ device, with a detection limit of 0.3–6 ppb (Varian, Belrose, Australia). Tin’s concentration was measured on an ICP OES Spectro Blue SOP, using a radial view into the plasma (SPECTRO Analytical Instruments, Kleve, Germany). Phase analysis was performed by the XRD method (X-ray diffraction phase analysis) with an X-ray powder diffractometer (Philips X’Pert PRO MRD (Co-Kα) and a measurement range of 10–120° 2 theta, with a scan step of 0.0170°) (Philips, Amsterdam, The Netherlands). The samples were prepared according to the standardised Panalytical backloading system, which provides an almost random distribution of particles. A scanning electron microscope MIRA3 FE-SEM (TESCAN, Prague, Czech Republic) was used to observe the morphology and determine the particle sizes; SEM also enabled a semi-quantitative elemental analysis via the EDX method. The EDX data were processed by the AZtec software, v5.0. (Oxford Instruments, Oxford, UK). The leachability test was performed according to the European standard STN EN 12457-1:2006 [40]. This standard is focused on the leachability test in the analysis of granular waste materials and sludges to determine the class of landfill where the given waste can be deposited. The particle morphology of the dust sample was observed by optical microscopy with a Dino-Lite ProAM413T digital microscope (AnMo Electronics Corporation, Hsinchu, Taiwan, with magnification under 100×) at a 50-fold magnification. The Pourbaix Eh–pH diagrams were used to evaluate the leaching thermodynamics using HSC Chemistry, version 10.0.2.3, with a University Basic licence (Metso Finland Oy, Espoo, Finland).

## 3. Results and Discussion

### 3.1. Sample Characterisation

**Figure 2:**
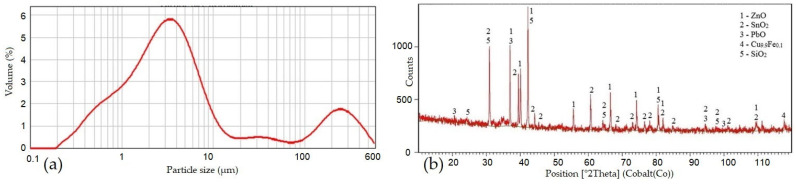
The distribution of the particle size of the CCFD sample (**a**); diffractogram from the phase analysis of the CCFD sample (**b**).

The weight loss of the representative sample by drying was 20.03%, and the weight loss by annealing was 40.55%. These values indicate a high content of moisture and chemically bound water; however, these values may also indicate the content of carbonates, chlorides, and volatile substances. It is possible for moisture to enter the converter flue dust due to the nature of the charge entering the converter, which includes not only the black copper (70 to 75% copper) that is the product of smelting in the shaft furnace but also scrap materials containing 60 to 85% copper [41]. From the course of the distribution curve shown in Figure 2a, the particle distribution is bimodal. A major part of the flue dust (77.5%) has a particle size from 0.399 μm to 11.247 μm. The average specific gravity of the converter flue dust from copper production is 3.4165 g × cm^−3^ with a standard deviation of 0.0071 g × cm^−3^. The flue dust is a fine dusty material.

The content of the major, minor, and trace elements of the sample of the converter flue dust, determined by the AAS method, is documented in Table 1. Zinc (38.31 wt.%), tin (2.92 wt.%), and lead (11.35 wt.%) have the highest representation. The flue dust also contains copper (0.71 wt.%), iron (0.13 wt.%), and nickel (0.05 wt.%) as minor elements. The determined trace element was cadmium, whose content in the flue dust is 0.009 wt.%. The chemical analysis shows that the flue dust represents a valuable secondary raw material, especially that of zinc, tin, lead, and copper. For a comparison with primary raw materials, it can be stated that copper ore usually contains 0.5 to 2% Cu [42]; commercially important lead ores typically contain 3 to 10% [43], averaging 3.4% Pb [44]; zinc ores have from 5 to 15% Zn [45]; lead–zinc ores typically contain 1 to 5% Pb and 1 to 10% Zn [46]; granite rock, with which Sn minerals are often associated, contains 1% tin oxide, but most tin is produced from sedimentary deposits containing 0.015% Sn [46].

From the results of the phase analysis (Figure 2b, Table 2), it is known that the flue dust consists mainly of metal oxides ZnO, SnO_2_, and PbO, the source of which is the metal-bearing part of the charge. There is also SiO_2_ in the dust, which probably comes from slag-forming additives and lining. In addition, according to the phase analysis, the flue dust also contains Cu_9.9_Fe_0.1_ alloy. In phase analysis, the flue dust did not swell, indicating that it is not hygroscopic.

**Figure 3:**
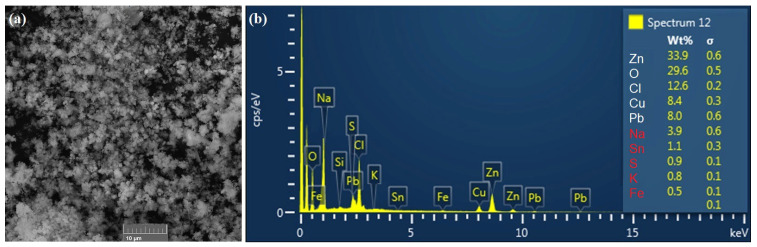
Results of SEM analysis of the flue dust sample (**a**); EDX analysis of the flue dust sample (**b**).

The morphology of the particles of the flue dust sample made by the SEM method is documented in Figure 3a, and in Figure 3b is the result of EDX analysis. The flue dust has a light beige-grey colour and is made up of fine spherical or almost spherical particles that form clusters in places. The results of the surface EDX analysis show similar values as the flue dust composition determined by the AAS method. The major elements are zinc, tin, and lead. The presence of copper, nickel, and iron was confirmed in smaller quantities. The above implies the presence of metals in the form of oxides.

**Figure 4:**
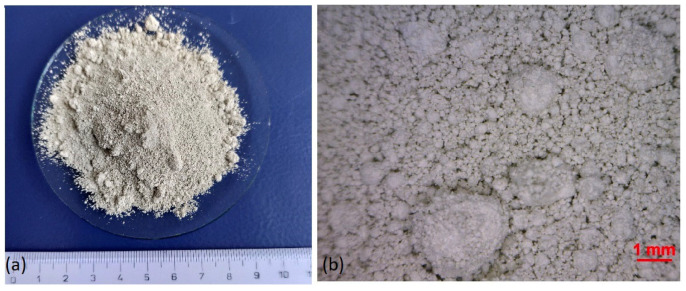
The CFD particles (without magnification) (**a**); Morphology of CFD particles sample under stereo microscopy (**b**).

A view of the flue dust particles (without magnification) is in Figure 4a, and at a 50-fold magnification, it is shown in Figure 4b.

The converter flue dust is formed by spherical or almost spherical particles of beige-grey colour. The sample consisted of two fractions, a coarse and a fine fraction. Larger-sized grains were covered by smaller grains. The smooth surface of the particles can reduce the reactivity of leaching the flue dust. From the results of the leachability test shown in Table 3, it was found that the limit values for the concentration of lead, tin, and zinc in the leachate, as well as the pH value, were exceeded. According to the decree of the Ministry of the Environment of the Slovak Republic, no. 382/2018 Coll., the concentration limit values for leachability class II (i.e., for landfilling in a landfill for non-hazardous waste) of lead, tin, and zinc in the leachate are 1 mg/L, 5 mg/L, and 5 mg/L in the given order, and the limit value of the pH is 6–12. From the decree of the Ministry of the Environment of the Slovak Republic, no. 382/2018 Coll., the concentration limit values for leachability class III (i.e., for disposal in a hazardous waste landfill) of lead, tin, and zinc in the leachate are 5 mg/L, 20 mg/L, and 20 mg/L, respectively, and the pH limit value is 5.5–13. This flue dust can be landfilled only at a hazardous waste landfill. According to the Regulation of the Government of the Slovak Republic no. 212/2022 Coll., the current fee rate for landfilling hazardous industrial waste is 40 euros per ton of waste for one calendar year.

The study of thermodynamics is necessary for the design of suitable processing of any material. The subject of the work is the processing of the converter flue dust from the secondary production of copper; therefore, the next chapter will refer to the thermodynamic study of the leaching of the converter flue dust in individual leaching agents.

### 3.2. Study of Thermodynamics of Leaching of the Converter Flue Dust in Individual Leaching Agents

The HSC Chemistry program, version 10.0.2.3, was used to evaluate the thermodynamics of leaching CCFD. HSC Chemistry is thermochemical equilibrium calculation software based on Gibbs free energy minimization, containing various databases [47]. This evaluation took place according to the following procedure. The phase composition of the given flue dust shows the presence of lead, zinc, and tin elements in the form of PbO, ZnO, and SnO_2_ oxides. A given Eh–pH diagram of the element and leaching medium at 20 °C (ambient temperature) and 80 °C (elevated temperature) was constructed to determine which of the given temperatures was more appropriate. From the literature review, leaching agents were selected for thermodynamic study. When the leaching medium was an acid solution (CH_3_COOH, H_2_SO_4_, or HNO_3_), the pH was set in the range 0–7. When the leaching medium was a base solution (NaOH), the pH was set in the range 7–14. The molality of both the major and minor elements was kept at a value of 1 mol/kg H_2_O, and the pressure remained at 1 bar (100 kPa). The form of the presence of elements (Pb, Zn, and Sn) in the area of water stability was determined from the constructed Eh–pH diagrams. According to the form of lead, zinc, and tin in the area of water stability, the chemical equation was then completed. The chemical equation was balanced, and the values of Gibbs free energy (ΔG°_T_) were calculated for the temperatures 20 to 90 °C. Graphs of ΔG°_T_ dependence of individual equations on temperature were created. Based on the graphs of the ΔG°_T_ dependence of individual equations on temperature and Eh–pH diagrams, the results obtained by the thermodynamic study were evaluated.

#### 3.2.1. Leaching of PbO, ZnO, and SnO_2_ in CH_3_COOH Solution

**Figure 5:**
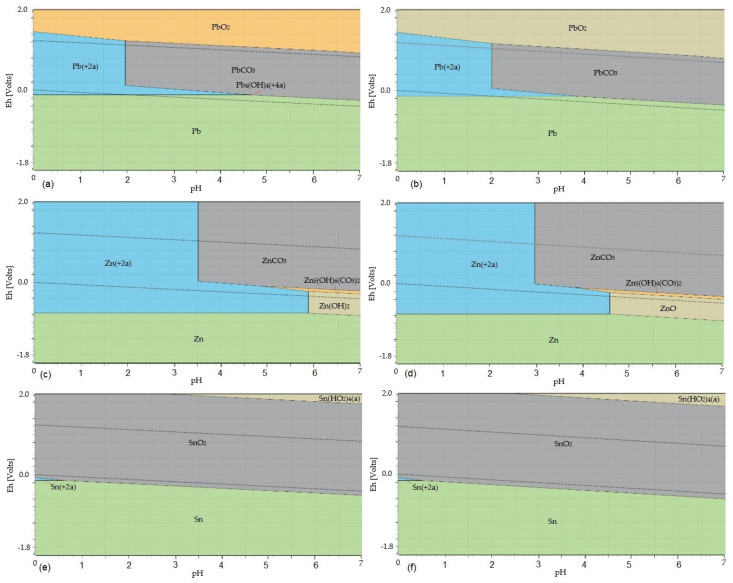
Eh–pH diagrams of leaching of PbO, ZnO, and SnO_2_ in CH_3_COOH at temperatures of 20 °C and 80 °C: (**a**) the Pb–C–H_2_O system at a temperature of 20 °C; (**b**) the Pb–C–H_2_O system at a temperature of 80 °C; (**c**) the Zn–C–H_2_O system at a temperature of 20 °C; (**d**) the Zn–C–H_2_O system at a temperature of 80 °C; (**e**) the Sn–C–H_2_O system at a temperature of 20 °C; (**f**) the Sn–C–H_2_O system at a temperature of 80 °C. Note: The dotted lines mark the water stability boundaries.

Figure 5 shows the potential–pH diagrams of PbO, ZnO, and SnO_2_ leaching in a CH_3_COOH solution at temperatures of 20 °C and 80 °C. The leaching Equations (36)–(38) are considered, given their values of Gibbs free energy at individual temperatures in Table 4.

**Figure 6:**
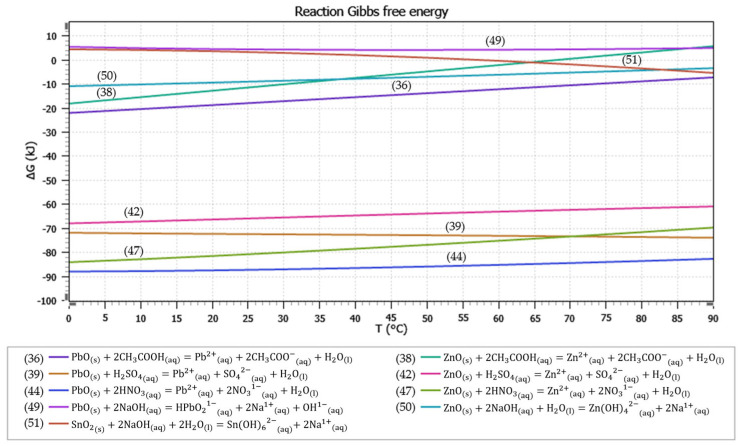
Graph of dependence of ΔG°_T_ values of Equations (36), (38), (39), (42), (44), (47), (49), (50), and (51) on temperature.

The Eh–pH diagrams shown in Figure 6 show that lead is present in the solution only as Pb^2+^_(aq)_ at a temperature of 20 °C in the pH range 0–1.95 and at a temperature of 80 °C at a pH of 0–2; zinc goes into the solution only in the form of Zn^2+^_(aq)_ at a temperature of 20 °C at a pH of 0–3.5 and at a temperature of 80 °C at a pH of 0–2.95; tin remains at both temperatures 20 °C and 80 °C throughout the whole acidic pH range present as SnO_2(s)_, not going into solution.

Figure 6 is a visual comparison of the ΔG°_T_ values of the considered leaching equations as a function of temperature.

From the comparison of the ΔG°_T_ values of Equations (36) and (38) shown in the graph in Figure 6, it follows that Equation (36) has a higher probability of proceeding in the direction of the formation of products. Equation (36) will take place up to a temperature of 90 °C, and Equation (38) only up to a temperature of 60 °C. The ΔG°_T_ values of Equations (36) and (38) increase with temperature; therefore, a lower temperature is more suitable for the leaching process of the flue dust in acetic acid solution. Using ambient temperature instead of an elevated temperature also saves operating costs.

#### 3.2.2. Leaching of PbO, ZnO, and SnO_2_ in H_2_SO_4_ Solution

**Figure 7:**
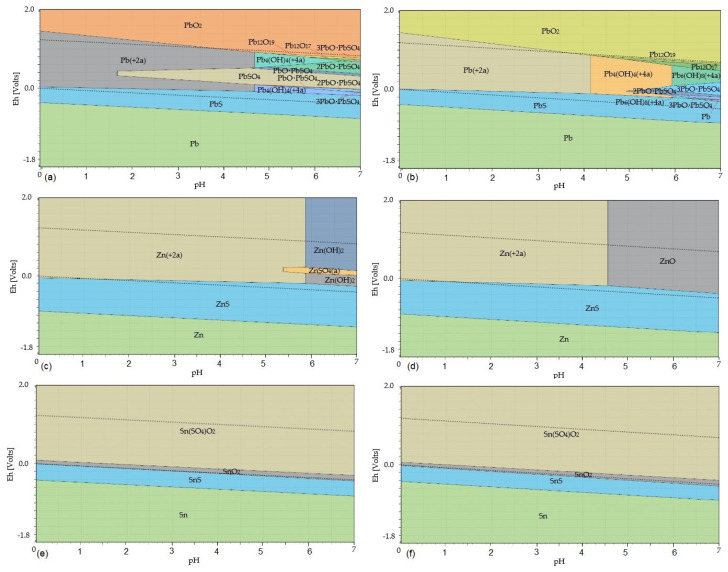
Eh–pH diagrams of leaching of PbO, ZnO, and SnO_2_ in H_2_SO_4_ at temperatures of 20 °C and 80 °C: (**a**) the Pb–S–H_2_O system at a temperature of 20 °C; (**b**) the Pb–S–H_2_O system at a temperature of 80 °C; (**c**) the Zn–S–H_2_O system at a temperature of 20 °C; (**d**) the Zn–S–H_2_O system at a temperature of 80 °C; (**e**) the Sn–S–H_2_O system at a temperature of 20 °C; (**f**) the Sn–S–H_2_O system at a temperature of 80 °C. Note: The dotted lines mark the water stability boundaries.

Figure 7 shows the potential–pH diagrams of PbO, ZnO, and SnO_2_ leaching in H_2_SO_4_ solution at temperatures of 20 °C and 80 °C.

The Eh–pH diagrams shown in Figure 7 show that lead is present in the solution only as Pb^2+^_(aq)_ at a temperature of 20 °C in the pH range 0–1.65; at a higher pH, other forms of lead are formed. At a temperature of 80 °C, lead is present in the solution only as Pb^2+^_(aq)_ at pH 0–4.5. Zinc enters the solution only in the form of Zn^2+^_(aq)_ at a temperature of 20 °C at a pH of 0–5.4; at a pH above 5.4, ZnSO_4(aq)_ is formed. At a temperature of 80 °C and a pH of 0–4.6, Zn^2+^_(aq)_ is formed in the solution. Tin remains at both temperatures of 20 °C and 80 °C throughout the acidic pH range, present as SnO_2(s)_ and Sn(SO_4_)O_2(s)_, and does not enter the solution. Leaching of the flue dust in H_2_SO_4_ can be described by Equations (39)–(43), whose values of Gibbs free energy at temperatures of 20 °C and 80 °C are given in Table 4. From the comparison of the ΔG°_T_ values of Equations (39) and (42) shown in Figure 6, it follows that Equation (39) has a higher probability of proceeding in the direction of the formation of products compared to Equation (42). The ΔG°_T_ values of reaction (39) decrease with temperature, and the ΔG°_T_ values of Equation (42) increase with temperature. A lower temperature is more suitable for the leaching process in sulphuric acid solution. Also, by using the ambient temperature, operating costs are saved.

#### 3.2.3. Leaching of PbO, ZnO, and SnO_2_ in HNO_3_ Solution

**Figure 8:**
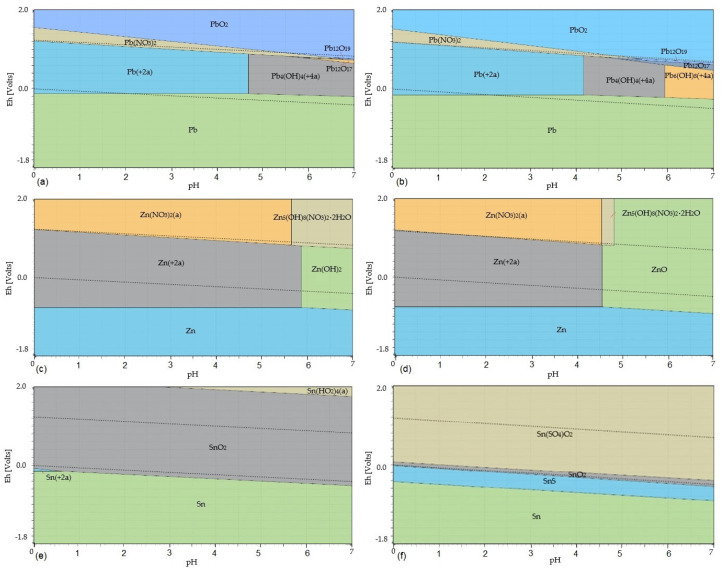
Eh–pH diagrams of leaching of PbO, ZnO, and SnO_2_ in HNO_3_ at temperatures of 20 °C and 80 °C: (**a**) the Pb–N–H_2_O system at a temperature of 20 °C; (**b**) the Pb–N–H_2_O system at a temperature of 80 °C; (**c**) the Zn–N–H_2_O system at a temperature of 20 °C; (**d**) the Zn–N–H_2_O system at a temperature of 80 °C; (**e**) the Sn–N–H_2_O system at a temperature of 20 °C; (**f**) the Sn–N–H_2_O system at a temperature of 80 °C. Note: The dotted lines mark the water stability boundaries.

Figure 8 shows the potential–pH diagrams of PbO, ZnO, and SnO_2_ leaching in HNO_3_ solution at temperatures of 20 °C and 80 °C.

Equations (44)–(48) describe the leaching of the flue dust in the HNO_3_ solution. Equations (44)–(48), with their values of Gibbs free energy at selected temperatures, are listed in Table 4. The Eh–pH diagrams shown in Figure 8 show that lead is present in the solution as Pb^2+^_(aq)_ at a temperature of 20 °C in the pH range of 0–4.7; at a higher pH, Pb_4_(OH)_4_^4+^_(aq)_ is formed. At a temperature of 80 °C, lead is present in the solution as Pb^2+^_(aq)_ at a pH of 0–4.7, at a pH of 4.7–7 as Pb_4_(OH)_4_^4+^_(aq)_, and at a pH of 5.9–7 as Pb_6_(OH)_8_^4+^_(aq)_. Zinc goes into the solution only in the form of Zn^2+^_(aq)_ at a temperature of 20 °C at a pH of 0–5.9. At a temperature of 80 °C and a pH of 0–4.6, Zn^2+^_(aq)_ is formed in the solution. Tin remains at both 20 °C and 80 °C throughout the whole acidic pH range present in the solid residue as SnO_2(s)_ and does not enter the solution. From the comparison of the ΔG°_T_ values of Equations (44) and (47) shown in Figure 6, it follows that Equation (44) has a higher probability of proceeding in the direction of product formation compared to Equation (47). The ΔG°_T_ values of Equations (44) and (47) increase with temperature. Therefore, a lower temperature is more suitable for the leaching of the flue dust in nitric acid solution. Also, by using the ambient temperature, operating costs can be saved.

#### 3.2.4. Leaching of PbO, ZnO, and SnO_2_ in NaOH Solution

**Figure 9:**
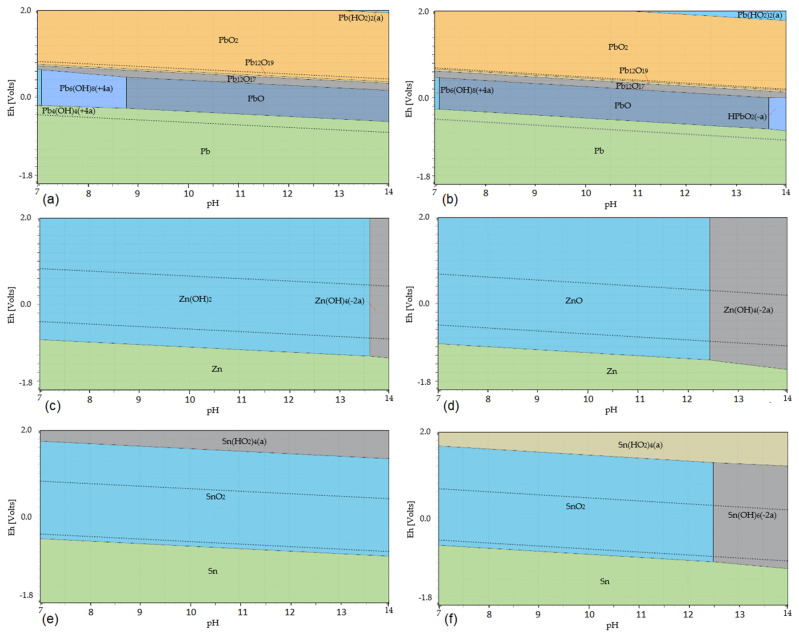
Eh–pH diagrams of leaching of PbO, ZnO, and SnO_2_ in NaOH at temperatures of 20 °C and 80 °C: (**a**) the Pb–Na–H_2_O system at a temperature of 20 °C; (**b**) the Pb–Na–H_2_O system at a temperature of 80 °C; (**c**) the Zn–Na–H_2_O system at a temperature of 20 °C; (**d**) the Zn–Na–H_2_O system at a temperature of 80 °C; (**e**) the Sn–Na–H_2_O system at a temperature of 20 °C; (**f**) the Sn–Na–H_2_O system at a temperature of 80 °C. Note: The dotted lines mark the water stability boundaries.

Figure 9 shows the potential–pH diagrams of PbO, ZnO, and SnO_2_ leaching in NaOH solution at temperatures of 20 °C and 80 °C. 

Equations (49)–(51) describe the leaching of flue dust in the NaOH solution. Equations (49)–(51), with their values of Gibbs free energy at temperatures of 20 °C and 80 °C, are listed in Table 4. The Eh–pH diagrams shown in Figure 9 show that lead is present in the solution as Pb_4_(OH)_4_^4+^_(aq)_ at a temperature of 20 °C in the pH range of 7–7.1 and at a pH of 7.1–8.8 as Pb_6_(OH)_8_^4+^_(aq)_. At a temperature of 80 °C, lead is present in the solution as Pb_6_(OH)_8_^4+^_(aq)_ at the pH range of 7–7.1 and at a pH range of 13.65–14 as HPbO_2_^1−^_(aq)_. Zinc enters the solution in the form of Zn(OH)_4_^2−^_(aq)_ at a temperature of 20 °C at a pH of 13.6–14. At the temperature of 80 °C and a pH of 12.45–14, Zn(OH)_4_^2−^_(aq)_ is formed in the solution. Tin remains at both 20 °C and 80 °C throughout the whole acidic pH range present in the solid residue as SnO_2(s)_ and does not enter the solution. From the comparison of the ΔG°_T_ values of Equations (49)–(51) shown in Figure 6, it follows that Equation (50) has the highest probability of proceeding in the direction of the formation of products at a temperature of 0–84 °C among the listed reactions. At a temperature above 84 °C, Equation (51) is most likely to occur. The ΔG°_T_ values of Equation (49) do not change significantly with temperature; they decrease up to a temperature of 45 °C and increase again from a temperature of 45 °C. The ΔG°_T_ values of Equation (50) increase with temperature, and the ΔG°_T_ values of Equation (51) decrease with temperature. A lower temperature is more suitable for the selective leaching of zinc from the dust in the NaOH solution. Moreover, by using the ambient temperature, operating costs are saved.

### 3.3. Evaluation of the Study of the Thermodynamics of Leaching of the Converter Flue Dust

The use of HNO_3_ and H_2_SO_4_ as leaching agents could produce toxic and irritating gases. In addition, leaching PbO in H_2_SO_4_ solution results in the formation of a PbSO_4_ precipitate according to Equation (52), given with G°_T_ values in Table 5.

The solubility product constant (K_sp_) of lead sulphate (PbSO_4_) at a temperature of 25 °C and an ionic strength of I = 0.0 mol dm^−3^ is 1.8 × 10^−8^. It is, therefore, a poorly soluble substance, where precipitation occurs to a large extent, and only a negligible amount of PbSO_4_ (5.46 × 10^−6^ g per litre of solution) remains dissolved in the solution at a temperature of 25 °C. According to the ΔG°_T_ values of Equation (52), the probability of PbSO_4_ precipitation should increase with temperature, while according to the Eh–pH diagrams shown in Figure 7, the formation of a PbSO_4_ precipitate should not occur at a temperature of 80 °C [48]. The NaOH solution is not selective enough since all three examined oxides would dissolve in the solution of NaOH, although tin was only at an elevated temperature (80 °C). Leaching in NaOH solution could be used to treat the solid residue from the first leaching. Also, leaching in H_2_SO_4_ solution is the most often used, and leaching in CH_3_COOH is not sufficiently investigated. For the above-mentioned reasons, the CH_3_COOH solution appears to be a suitable proposed selective leaching agent. The evaluation of the possibility of leaching the major components of the flue dust in individual leaching media is summarised in Table 6.

After determining the leaching conditions, it is possible to propose a method of obtaining metals, namely lead and zinc. A suitable extraction method for obtaining lead from the leachate is the cementation method. A suitable lead cementer is zinc. This follows not only from the galvanic series (or electropotential series) since the electrode potential of the redox pair Zn^2+^/Zn^0^ is more negative compared to the electrode potential of the redox pair Pb^2+^/Pb^0^ (Equation (53)), but also because using zinc as a cementing metal would not pollute the solution since zinc is already present in the solution (Equation (54)) [49].

(53) $${E^{0}}_{{Zn}^{2+}/{Zn}^{0}}<{E^{0}}_{{Pb}^{2+}/{Pb}^{0}}\;\;\;-0.8V<-0.1V$$

(54) $${Zn}_{\left(s\right)}+{Pb{\left(CH_{3}CO_{2}\right)}_{2}}_{\left(aq\right)}={Zn{\left(CH_{3}COO\right)}_{2}}_{\left(aq\right)}+{Pb}_{\left(s\right)}∆G_{20\;{}^{\circ}C}=-143.155\;kJ$$

After removing the lead from the solution, the zinc can be obtained by the precipitation method. Suitable precipitation agents are carbonate ions in the form of, e.g., ammonium carbonate ((NH_4_)_2_CO_3_) or sodium carbonate (Na_2_CO_3_). The general reaction of zinc carbonate precipitation is Equation (55). The value of the solubility product constant (K_sp_) of zinc carbonate (ZnCO_3_) at a temperature of 25 °C and ionic strength of I = 0.0 mol dm^−3^ is 1.2 × 10^−10^.

(55) $${{Zn}^{2+}}_{\left(aq\right)}+{{CO_{3}}^{2-}}_{\left(aq\right)}={ZnCO_{3}}_{\left(s\right)}\;\;\;∆G_{20\;{}^{\circ}C}=-55.124\;kJ$$

## 4. Conclusions

This study investigated treatments of the CCFD as a metal-bearing waste generated during metal production from a copper recycling company. It is extremely important to find the right processing technologies so that metals, as valuable raw materials, are not lost. Crucial factors influencing the direction of development of processing technologies are the economy of the process and the impact on the environment. In the scientific literature, there are several research articles on the processing of copper flue dusts. A suitable method for processing CCFD appears to be hydrometallurgy. The possibility of leaching major components of the flue dust in selected leaching agents was confirmed or disproved by a thermodynamic study using Eh–pH diagrams for individual metal–S/C/N/Na/–H_2_O systems. Subsequently, treatment of the leachate and the solid residue were proposed. The results are as follows:

(1) The converter flue dust contains a considerable proportion of zinc, lead, and tin. These metals are present in the flue dust in the form of oxides, such as ZnO, PbO, and SnO_2_. The size distribution of the fine-grained flue dust particles has a bimodal character. The size of particles is mostly in the range below 11.247 μm. The flue dust consists of spherical or almost spherical particles of beige-grey colour. Flue dust from copper production is hazardous waste, but it also represents a valuable secondary raw material.
(2) From a theoretical overview of the possibilities of processing flue dust by a hydrometallurgical method, a solution of acetic acid is a suitable selective leaching agent. The use of aqueous solutions of HNO_3_ and H_2_SO_4_ as leaching agents can produce toxic and irritating gases, which is disadvantageous from an environmental point of view. Furthermore, leaching PbO in a H_2_SO_4_ solution causes PbSO_4_ precipitation. The NaOH solution is not selective enough since all three investigated oxides dissolve in it, although tin can only be dissolved at elevated temperatures (80 °C). When leaching in an acetic acid solution, zinc and lead are chemically dissolved to form acetates Zn(CH_3_COO)_2_ and Pb(CH_3_COO)_2_, while tin remains in the form of SnO_2_ in the solid residue. Leaching of CCFD in an acetic acid solution is also effectively conducted at an ambient temperature and at a pH of 0–1.95, which is also advantageous from an economic point of view.
(3) After leaching, the extraction method of zinc cementation of lead was proposed. Precipitation of zinc from the solution after cementation is conducted by carbonate ions. Since the tin remains in the solid residue after leaching, the solid residue could be subsequently processed in the second stage of leaching in an alkaline NaOH solution.

Optimal conditions of leaching and extraction methods, kinetics study, and product characterisation may be the subject of future research.

## Figures and Tables

**Table 1:** Elemental composition of the converter flue dust sample.

Content [wt.%]	Zn	Pb	Sn	Fe	Cu	Ni	Cl^−^
Average	38.31	11.35	2.92	0.13	0.71	0.05	30.58
Standard deviation	2.83	0.96	0.06	0.09	0.02	6.9 × 10^−18^	3.51
Variance	8.02	0.93	0.00	0.00	0.00	4.8 × 10^−35^	12.37

**Table 2:** List of phases present in the flue dust sample.

Ref. Code	Compound Name	Chemical Formula
01-079-0208	Zinc Oxide	ZnO
01-077-0452	Tin Oxide	SnO_2_
01-085-1289	Lead Oxide	PbO
00-040-1294	Copper Iron	Cu_9.9_Fe_0.1_
01-089-8938	Silicon Oxide	SiO_2_

**Table 3:** The results of the leachability test of dust according to the decree of the Ministry of the Environment of the Slovak Republic, no. 382/2018 Coll.

Monitored Parameter	pH	Concentration (mg/L)										
		As	Co	Cr	Cu	Ni	Pb	Sn	Zn	Sb	Cd	Cl^−^
Leachate	5.5	0	0	0	0	0.23	23.64	14.5	15.6	0	0	710

**Table 4:** Values of Gibbs free energy of Equations (36)–(51).

Chemical Reaction	ΔG°_T_ [kJ/mol]		No.
	20 °C	80 °C	
${PbO}_{\left(s\right)}+2{CH_{3}COOH}_{\left(aq\right)}={{Pb}^{2+}}_{\left(aq\right)}+2{{CH_{3}COO}^{-}}_{\left(aq\right)}+{H_{2}O}_{\left(l\right)}$	−18.927	−9.041	(36)
$4{PbO}_{\left(s\right)}+4{CH_{3}COOH}_{\left(aq\right)}={{{Pb}_{4}{\left(OH\right)}_{4}}^{4+}}_{\left(aq\right)}+4{{CH_{3}COO}^{-}}_{\left(aq\right)}$	−74.577	−48.897	(37)
${ZnO}_{\left(s\right)}+2{CH_{3}COOH}_{\left(aq\right)}={{Zn}^{2+}}_{\left(aq\right)}+2{{CH_{3}COO}^{-}}_{\left(aq\right)}+{H_{2}O}_{\left(l\right)}$	−12.947	+2.968	(38)
${PbO}_{\left(s\right)}+{H_{2}SO_{4}}_{\left(aq\right)}={{Pb}^{2+}}_{\left(aq\right)}+{{SO_{4}}^{2-}}_{\left(aq\right)}+{H_{2}O}_{\left(l\right)}$	−72.478	−73.772	(39)
$4{PbO}_{\left(s\right)}+2{H_{2}SO_{4}}_{\left(aq\right)}={{{Pb}_{4}{\left(OH\right)}_{4}}^{4+}}_{\left(aq\right)}+2{{SO_{4}}^{2-}}_{\left(aq\right)}$	−181.679	−178.359	(40)
$6{PbO}_{\left(s\right)}+2{H_{2}SO_{4}}_{\left(aq\right)}+2{H_{2}O}_{\left(l\right)}={{{Pb}_{6}{\left(OH\right)}_{8}}^{4+}}_{\left(aq\right)}+2{{SO_{4}}^{2-}}_{\left(aq\right)}$	−193.948	−188.027	(41)
${ZnO}_{\left(s\right)}+{H_{2}SO_{4}}_{\left(aq\right)}={{Zn}^{2+}}_{\left(aq\right)}+{{SO_{4}}^{2-}}_{\left(aq\right)}+{H_{2}O}_{\left(l\right)}$	−66.498	−61.763	(42)
${ZnO}_{\left(s\right)}+{H_{2}SO_{4}}_{\left(aq\right)}={ZnSO_{4}}_{\left(aq\right)}+{H_{2}O}_{\left(l\right)}$	−79.306	−81.503	(43)
${PbO}_{\left(s\right)}+2{HNO_{3}}_{\left(aq\right)}={{Pb}^{2+}}_{\left(aq\right)}+2{{NO_{3}}^{1-}}_{\left(aq\right)}+{H_{2}O}_{\left(l\right)}$	−87.662	−83.770	(44)
$4{PbO}_{\left(s\right)}+4{HNO_{3}}_{\left(aq\right)}={{{Pb}_{4}{\left(OH\right)}_{4}}^{4+}}_{\left(aq\right)}+4{{NO_{3}}^{1-}}_{\left(aq\right)}$	−212.046	−198.354	(45)
$6{PbO}_{\left(s\right)}+4{HNO_{3}}_{\left(aq\right)}+2{H_{2}O}_{\left(l\right)}={{{Pb}_{6}{\left(OH\right)}_{8}}^{4+}}_{\left(aq\right)}+4{{NO_{3}}^{1-}}_{\left(aq\right)}$	−224.315	−208.023	(46)
${ZnO}_{\left(s\right)}+2{HNO_{3}}_{\left(aq\right)}={{Zn}^{2+}}_{\left(aq\right)}+2{{NO_{3}}^{1-}}_{\left(aq\right)}+{H_{2}O}_{\left(l\right)}$	−81.682	−71.761	(47)
${ZnO}_{\left(s\right)}+2{HNO_{3}}_{\left(aq\right)}={Zn{\left(NO_{3}\right)}_{2}}_{\left(aq\right)}+{H_{2}O}_{\left(l\right)}$	−82.324	−72.293	(48)
${PbO}_{\left(s\right)}+2{NaOH}_{\left(aq\right)}={{HPbO_{2}}^{1-}}_{\left(aq\right)}+2{{Na}^{1+}}_{\left(aq\right)}+{{OH}^{1-}}_{\left(aq\right)}$	+4.412	+4.517	(49)
${ZnO}_{\left(s\right)}+2{NaOH}_{\left(aq\right)}+{H_{2}O}_{\left(l\right)}={{Zn{\left(OH\right)}_{4}}^{2-}}_{\left(aq\right)}+2{{Na}^{1+}}_{\left(aq\right)}$	−9.559	−4.485	(50)
${SnO_{2}}_{\left(s\right)}+2{NaOH}_{\left(aq\right)}+2{H_{2}O}_{\left(l\right)}={{Sn{\left(OH\right)}_{6}}^{2-}}_{\left(aq\right)}+2{{Na}^{1+}}_{\left(aq\right)}$	+3.509	−3.642	(51)

**Table 5:** Values of Gibbs free energy of Equation (52).

Chemical Reaction	ΔG°_T_ [kJ/mol]		No.
	20 °C	80 °C	
${PbO}_{\left(s\right)}+{H_{2}SO_{4}}_{\left(aq\right)}={PbSO_{4}}_{\left(s\right)}+{H_{2}O}_{\left(l\right)}$	−116.369	−125.669	(52)

**Table 6:** Evaluation of the leaching of PbO, ZnO, and SnO_2_ in individual leaching agents.

Oxide	Leaching Agent and Temperature							
	HNO_3_		NaOH		CH_3_COOH		H_2_SO_4_	
	20 °C	80 °C	20 °C	80 °C	20 °C	80 °C	20 °C	80 °C
PbO	+	+	+	+	+	+	−	−
ZnO	+	+	+	+	+	+	+	+
SnO_2_	−	−	−	+	−	−	−	−

Note: + leaching is in progress, − leaching is not in progress.

## Data Availability

The original contributions presented in the study are included in the article, further inquiries can be directed to the corresponding author.
